# (Holo-PACS): use of holograms to improve provider acumen when reading clinical radiographic scans

**DOI:** 10.1080/01616412.2026.2714537

**Published:** 2026-09-01

**Authors:** Brett C. Meyer, Ben Shifflett, Weichen Liu, Kunal Agrawal, Dawn M. Meyer, Reza Bavarsad Shahripour, Royya Modir, Thomas M. Hemmen, Sanad Batarseh, Marissa D’Souza, Tiffany Hu, Cattien Phan, Amy Radonich, Nadir Weibel

**Affiliations:** a Neurosciences, University of California San Diego, La Jolla, California, USA;; b Mathematics, University of California San Diego, La Jolla, California, USA;; c Imaging Informatics, University of California San Diego, La Jolla, California, USA;; d Computer Science and Engineering & Design Lab UC San Diego, University of California San Diego, La Jolla, California, USA

**Keywords:** Hologram, education, stroke, fellowship, CTA, augmented reality, satisfaction

## Abstract

**Background and purpose:**

Vascular Neurology (VN) fellowship education in CTA interpretation is critical, given the increased opportunities for tissue salvage via neurointervention. Holo-Stroke-CTA 3D visualization has already shown improved accuracy and satisfaction for vessel occlusion detection. Our aim in this present analysis was to determine if focused education in CTA reading, including addition of Holo-Stroke-CTA and immediate access to clinical PACS images during team meetings (Holo-PACS), would improve fellow and team education.

**Methods:**

Four VN fellows scored 3 sets of de-identified CTAs (DICOM Group-A (*n* = 12), DICOM Group-B (*n* = 12), and Holo-Stroke-CTA Group-C (*n* = 24) to assess for LVOs/MeVOs. After each set, a training session was given. Concurrently, Holo-PACS, accessing our enterprise PACS system via secure Intranet, was utilized in weekly team conferences. Likert surveys assessed satisfaction. Wilcoxon Signed Rank was used for non-parametric, and Exact Binomial Test was used for preference comparisons.

**Results:**

The overall correct percentage was 63% DICOM Group A vs. 97% Group C Holo-PACS. MCA (75%,94%), ICA (75%,100%), and Basilar (100%,100%) locations scored high, with marked improvement for ACA (13%,100%), and PCA (38%,94%). Likert satisfaction overall was 47% DICOM vs. 94% Holo-PACS. ‘Satisfaction’ scores were (55%,90%), ‘Immersion’ (34%,96%), ‘Ease of Use’ (53%,87%), ‘Accuracy’ (55%,93%), ‘Technology Advancement’ (42%,98%), and ‘Enthusiasm’ (45%,97%).

**Conclusions:**

The Holo-PACS initiative, including focused CTA education and implementing both Holo-Stroke-CTA and secure connection to PACS imaging using Holo-PACS during weekly team meetings, resulted in an improved ability to interpret CTAs and robust immersive satisfaction. Holo-PACS is becoming integral to our stroke education and training, and may serve as a model for vascular neurology fellowship training programs.

## Introduction

In acute stroke, it is critical to evaluate for thrombolytic and neurointerventional (NIR) eligibility [1–8]. With the advent of NIR, the need to accurately assess for Large Vessel Occlusion (LVO) and Medium Vessel Occlusion (MeVO) has become critical [4–10]. Although neuroradiologists receive dedicated training in detecting occlusions, medical stroke specialists are often trained in an ‘apprenticeship-driven’, hands-on training program. Other providers who may not have completed a stroke fellowship may not receive focused education on occlusion detection.

Although assessing for middle cerebral artery (MCA) LVOs on Digital Imaging and Communications in Medicine (DICOM) imaging is commonly considered, many occlusions in other vessels are more difficult to find [11–17]. This becomes even more critical in telestroke. Telestroke programs often serve smaller hospitals with limited neuroradiology resources, resulting in these providers frequently interpreting their own Computed Tomography Angiography (CTA) images acutely [18,19].

Current state includes utilizing standard DICOM radiology tools to scroll through axial, coronal, and sagittal 2D radiology images. Although some DICOM viewers have tools to reformat into a 3D vessel view, this still only results in a 3D rotation appearance on a 2D computer monitor.

Utilizing a tool that will augment immediate CTA vessel LVO interpretation is beneficial for LVO and MeVO detection [20]. Incorporating Augmented Reality (AR) and 3D hologram technology (Holo-Stroke) into provider education for those desiring advanced stroke training is not yet broadly available [21]. Our goals were to both determine whether targeted CTA education early in fellowship improves LVO/MeVO detection accuracy, and to determine whether AR-based hologram viewing (Holo-PACS) further enhances user-reported experience compared to standard DICOM review.

Our Holo-PACS training and educational initiative does not exist in isolation. The use of augmented or mixed reality in medicine or surgery is not new. Prior studies have detailed mixed reality platforms for medical education, including bedside teaching, anatomy instruction, and neurosurgical holographic viewing for surgical planning [22–24]. Few studies, however, have specifically addressed CTA interpretation or vascular neurology (VN) fellowship education.

The aim of this analysis was not intended to show that Holo-PACS imaging alone was superior to standard DICOM imaging review. The aims were to highlight both the need for dedicated imaging review education in VN training, as well as point out how this education which necessarily includes review of numerous DICOM images using standard technology, can be supplemented with the 3D visualization technique of Holo-PACS.

## Materials and methods

### Quality improvement (QI) approval

This project was approved as a QI initiative (#1423). Although the Holo-PACS pathway was used for educational purposes, it was not used for clinical diagnosis.

### Setting and participants

The Holo-PACS technique/pilot program was incorporated into the education and training of our Accreditation Council for Graduate Medical Education (ACGME) accredited VN Stroke Fellowship to improve the quality of radiographic image assessment. Each year, our program educates VN Fellows on all aspects of stroke management and treatment. One important technique is the identification of LVOs on CTA. In acute patient evaluations, fellows become proficient at assessing for LVOs. Further, each week our stroke team members (6 faculty, 4 fellows, 1–2 nurse practitioners, and 2–4 coordinators) join a weekly clinical team meeting to review all cases of acute stroke from the prior week, which includes review of relevant CTAs.

### Datasets and case selection

For the direct training portion of this initiative, we first de-identified DICOM images that were drawn from acute stroke code cases, and included them in de-identified training datasets (Group-A (DICOM *n* = 12) and Group-B (DICOM *n* = 12)). Cases included LVO/MeVO (MCA, anterior cerebral artery (ACA), posterior cerebral artery (PCA), Basilar, and intracranial internal carotid artery (ICA)) and normal studies. DICOM cases were also rendered using the Holo-Stroke software and used for Group-C assessments (*n* = 24). Separately, for our weekly conferences, non-de-identified current-week cases were accessed via the enterprise PACS system on the secure intranet for educational review only.

### Holo-stroke-CTA

Our previously published Holo-Stroke-CTA system allows providers to render virtual 3D images in their physical space [20,25–28]. Users can rotate and re-size the image, and move around the vessels as if the rendering were present in their actual physical environment. This ‘at-a-glance’ visualization of the 3D vasculature allowed providers to more accurately detect LVOs. In prior assessments of VN Fellows, detection of LVO improved significantly (MCA improvement from 79% to 98%, ACA from 8% to 100%, PCA from 63% to 100%, and Basilar from 85% to 100%).

### Education pathway

The educational intervention was not a formal didactic lecture series but more so a multi-case review focusing on specific techniques and practical tips in vessel assessment. The educational objectives were clear and stated to the fellows at the onset of training. The goal was to impart as many ‘practical’ pearls as possible to rapidly right-shift the fellows’ knowledge base. This design was grounded in pragmatic recommendations due to the critical importance of being able to determine the presence of vascular occlusions in today’s NIR treatment paradigms. The faculty providing education has over a decade of experience training medical students in the pre-hospital neuroscience curriculum, has trained over 50 VN Fellows in clinical stroke management, and has greater than 25 years’ experience as a vascular neurology faculty member. Each approximately 1-hour session consisted of a personal review of each of the evaluation cases. No slides were used. Cases were chosen because they represented both the common, and the less common but critical (e.g. basilar occlusions), vessel occlusions that fellows would encounter. Using a standard DICOM approach, the faculty would identify the vessel and occlusion, pay special attention to various options for appropriate windowing and leveling for optimal view, review an approach to find and track each vessel from proximal to distal locations, and emphasize scenarios where various imaging orientations could be more helpful (e.g. using coronal imaging to help assess more distal MCA M2 trunks). Frequently encountered mistakes and pitfalls (e.g. mis-identifying veins as arteries or failing to assess specific locations for occlusion) would also be reviewed. A similar review was done after the Hologram evaluation but using the virtual hologram tools and techniques. Identical instruction was delivered to all fellows simultaneously, as each of the 4 VN fellows joined each of the 3 training sessions together. Fellows were encouraged to ask as many questions about each case as they desired.

Evaluation 1 was done after the first month of their stroke fellowship to assess base knowledge of VN Fellows early in fellowship. VN Fellows viewed and scored a de-identified case set (Group A) for LVO/MeVO using a standard DICOM solution. Scores were compared to the gold standard (the official neuroradiology interpretation). No clinical history was provided. After completion of Evaluation 1, the stroke faculty provided the focused CTA training session on how to read CTA images. Two weeks later, VN Fellows performed Evaluation 2 where they viewed and scored another de-identified case set (Group B) using a standard DICOM solution. After completion of Evaluation 2, the same stroke faculty provided another CTA training session reviewing results and providing additional guidance on reading CTA images for LVO detection. Two weeks later, fellows were then trained in viewing Holo-Stroke holograms. A 15-minute training session focused on use of the hologram software tool, and how to adjust and interact with the 3D hologram image. In Evaluation 3, VN Fellows assessed Group C images via Holo-PACS hologram. Holo-Stroke images only required 12 seconds to render from standard DICOM image files.

All scores were compared for reliability for multiple users using Kappa reliability statistics comparing each technique vs. gold standard based on Cohen’s Kappa [29]. Proportions of LVO presence/absence correct by group were compared using an overall four-sample proportion test then each combination was tested via pairwise proportion tests (holm correction applied). LVO proportions were compared between standard DICOM and Holo-Stroke-CTA using two-sample proportion test with continuity correction due to low sample size.

The faculty who originally collected and de-identified the images was charged with case allocation/distribution between groups to avoid an imbalance of case difficulty inadvertently affecting the results instead of the intended variable of training doing so. In an attempt to match diagnostic difficulty, the faculty first selected enough cases to result in an equal number of proximal (LVO) and distal (MeVO) occlusions for each vessel. Next, the faculty allocated equal amounts of LVOs and MeVOs for each vessel in question to each of the A, B, and C Groups. Within group order was then shuffled to avoid awareness as to which vessel occlusion would be presented next, and blind the VN reviewer to final case order. Reviewers were also not informed as to how many of each vessel LVO or MeVO would be included in each Group.

### Holo-PACS mechanism and information interaction flow

The training and subsequent clinical team education followed separate workflows, with the dedicated training sessions taking place over a single 4-week period. For those training sessions, the workflow included presenting a list of de-identified CTA images to each of the VN fellows for scoring and subsequent review.

**Figure 1.**
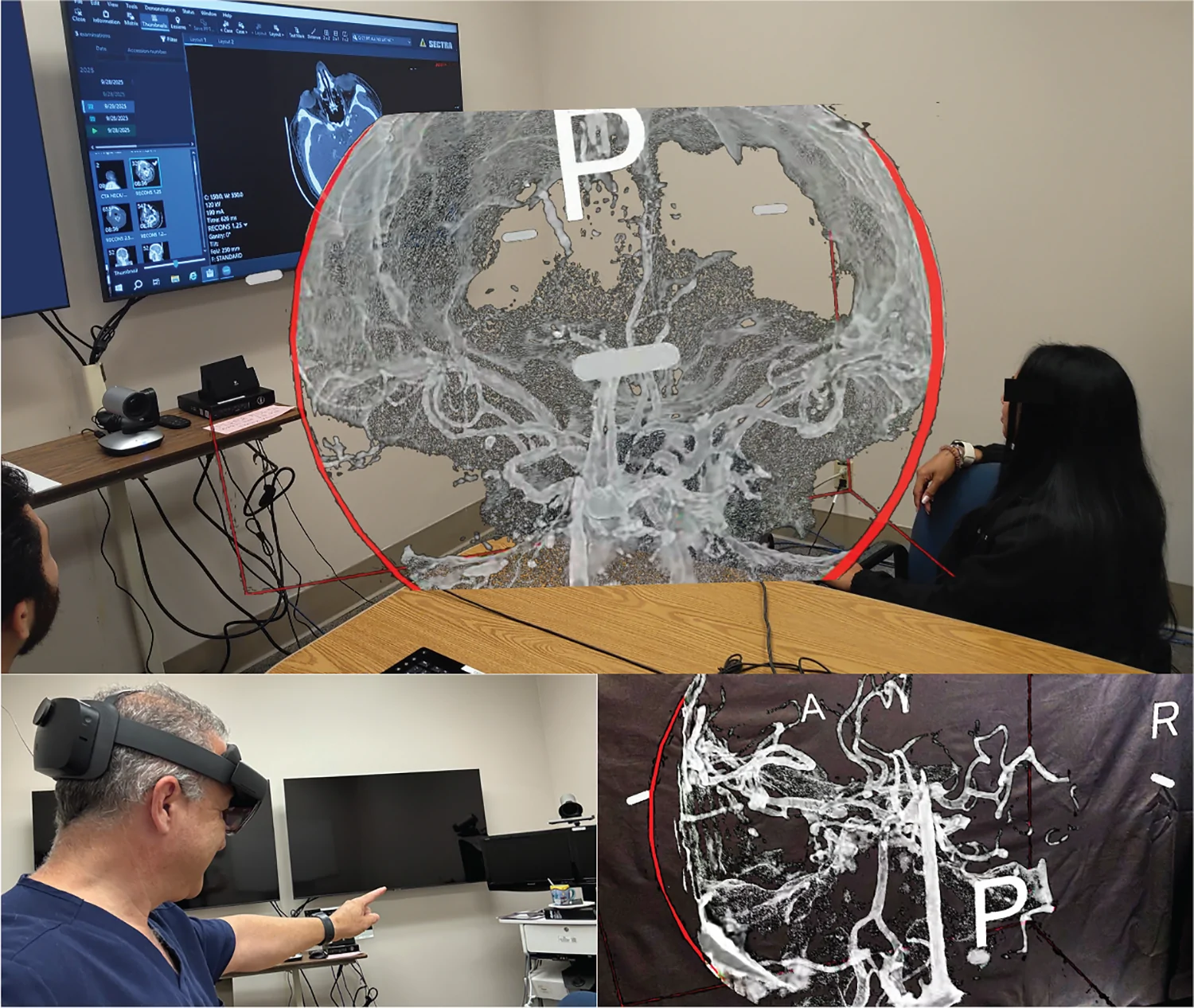
Holo-PACS set-up [Holo-PACS directory and CTA hologram views]. Figure 1 shows the Holo-PACS setup. (Top) CTA Hologram as viewed during weekly clinical case conferences, in stroke team conference room. This imaging is used for educational purposes to view 3D structure and LVOs, not for clinical diagnosis. (Bottom Left) View of provider wearing the HoloLens2 Head-Mounted-Display (HMD) and interacting with the Holo-Stroke-CTA hologram in the conference room. (Bottom Right) Holo-PACS Holo-Stroke CTA Hologram as rendered through the HoloLens2 viewer.

**Figure 2.**
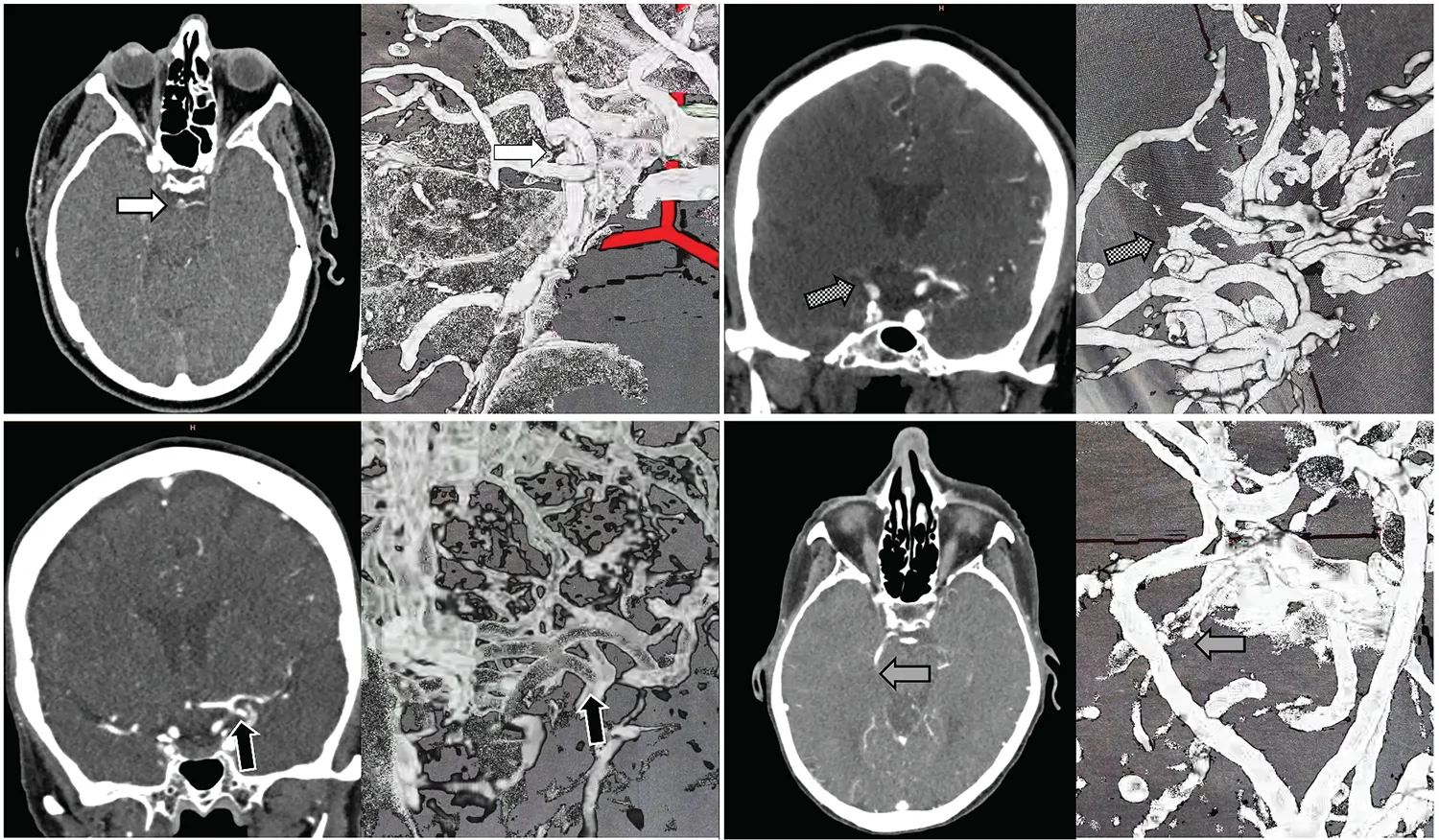
DICOM and Holo-Stroke-CTA views of challenging vessel occlusions. Figure 2 shows both DICOM and Holo-Stroke-CTA views of challenging vessel occlusions noted during this Holo-PACS experience. (Top-Left): White Arrow – Sub-occlusive thrombus in top of the Basilar artery. (Top-Right): Checkerboard Arrow – Right ICA occlusion. (Bottom-Left): Black Arrow – Duplicate Left MCA with inferior vessel occlusion. (Bottom-Right): Grey Arrow – Left PCA occlusion with overlying vein traversing in a similar path.

For the clinical team education workflow, the Holo-PACS pathway was used. Holo-PACS is an extension of Holo-Stroke-CTA for education. In the standing, weekly, clinical team meetings, cases are retrospectively reviewed from that prior week of clinical stroke codes. The standard enterprise PACS server is the repository for all clinical stroke code images. A subset of these images is routed to the Holo-PACS server via a securely networked filesystem, when an acute stroke code CTA order is tied to a CTA image. The server is accessed behind our own enterprise firewall (Intranet only) mitigating any risk to personal health information (PHI). This allows for immediate access and rendering of Hologram CTA images, and for the team to access these images at the time of our subsequent, weekly educational reviews. Holo-PACS access to the non-deidentified PACS server directory for hologram rendering is necessary for the continued clinical education portion of the Holo-PACS education pathway. Every week, the stroke team reviews difficult LVO (and other vascular) cases from the prior week via standard DICOM approach. At times, additional 3D views are incorporated by the team (and visualized by the Holo-PACS system using the HoloLens2 HMD) to gain additional perspectives (Figure 1). In our conference room, images are viewed in person, using the HoloLens to view the 3D hologram, or streamed using our HIPAA compliant software for all participants to see via videoconference what is being seen via the HMD. This tool is not used for clinical and diagnostic purposes at this time. Any diagnosis/radiographic interpretation has already been documented previously in official UCSD radiology reads. We may use this tool as a quality improvement/efficiency tool for looking at these images, as providers find it simpler to assess hologram images ‘at-a-glance’ instead of scrolling through 2D DICOM images using a standard DICOM viewer. Example Head CTA images (axial/coronal slices) and corresponding Hologram images showing arrows pointing to vessel occlusions are shown (Figure 2).

### Holo-PACS survey

Survey questions assessed overall satisfaction, efficiency, efficacy, enthusiasm, ease of use, effectiveness, preference, technological advancement, and future potential. Wilcoxon Signed Rank testing was used for non-parametric, matched pairs comparisons. Exact Binomial Test with Clopper-Pearson confidence intervals were used to compare which method (standard DICOM vs. Holo-PACS) providers preferred for each question and overall survey. The questionnaire was an investigator-developed, non-validated satisfaction survey previously used and intended for exploratory assessment of user perceptions. We utilized this prior survey as it has been used in our prior publication assessing the satisfaction of Holo-Stroke-CTA, and as it has demonstrated face validity.

## Results

Four VN Fellows, 1 month into Fellowship training, evaluated Group A (Standard DICOM CTAs), Group B (Standard DICOM CTAs) and Group C (Holo-Stroke/Holo-PACS CTAs). Reliability statistics (% correct and Kappa) for noting presence/absence of LVO are listed in Table 1. Fellows accurately noted presence/absence of LVO 69% (κ=0.28) of the time for Group A, 94% (κ=0.83) for Group B, and 99% (κ=0.94) of the time for Holo-Stroke Group C. Fellows accurately noted location of LVO 63% (κ=0.57) of the time for Group A, 83% (κ=0.81) for Group-B, and 98% (κ=0.96) of the time for Holo-Stroke Group C. Table 2 shows subset reliability analyses for assessing different vessels for LVO. Results for LVO detection are: ACA (13% for Group A, 63% for Group B, and 100% for Group C (κ=0.00, κ=0.50; κ=1.00)), MCA (75%, 100%, 94% (κ=0.50, κ=1.00; κ=0.75)), PCA (38%, 75%, 94% (κ=0.25, κ=0.67; κ=0.90)), ICA (75%, 88%, and 100% (κ=0.67, κ=0.83; κ=1.00)), Basilar (100%, 88%, and 100% (κ=1.00, κ=0.75; κ=1.00)), and Normal (75%, 88%, and 100% (κ=0.50, κ=0.75; κ=1.00)). A total of 13 stroke providers (6 faculty, 4 fellows, 2 NPs, and 1 coordinator) completed satisfaction surveys for the Holo-PACS process. Table 3 notes median Likert scale scores for all questions overall for DICOM vs. Holo-PACS, was 27 (IQR = 10) vs. 58 (IQR = 4) (*p* = 0.002), with overall percentage 47.44% vs. 93.59%. For the question related to ‘Satisfaction’, scores were 6 vs. 9 (*p* = 0.001), with overall percentage 55.38% vs. 90.00%. For ‘Immersion’, scores were 4 vs. 10 (*p* = 0.002), with overall percentage 33.85% vs. 96.15%. For ‘Ease of Use’, scores were 5 vs. 9 (*p* = 0.003), with overall percentage 53.08% vs. 86.92%. For ‘Accuracy’, scores were 5 vs. 9 (*p* = 0.002), with overall percentage 54.62% vs. 93.08%. For ‘Advanced Tech’, scores were 4 vs. 10 (*p* = 0.002), with overall percentage 42.31% vs. 98.46%. For ‘Interest/ Enthusiasm’, scores were 5 vs. 10 (*p* = 0.002), with overall percentage 45.38% vs. 96.92%. Comparative survey question results are also noted in Table 3 with overall median of 0 vs. 6 (*p* < 0.001), with overall percentage 3.58% vs. 96.15%.

## Discussion

The overall purpose of Holo-PACS was not simply to determine whether holographic CTA viewing improves the ability to ‘at-a-glance’ detect vessel occlusions. That was the focus of our prior Holo-Stroke-CTA publication [20]. Instead, the core innovation of Holo-PACS is the implementation of an educational initiative designed to improve the ability of the VN fellow to interpret CTAs. Our objective was to address this need for training in an era where rapid and accurate imaging interpretation is critical for neurointerventional therapies. Holo-PACS meets these aims both by standard DICOM review and Holo-Stroke-CTA visualization. We sought to determine whether this approach could improve the fellows’ ability to detect vascular occlusions, and provide an educational framework for augmenting fellowship training.

Brief targeted CTA education produced immediate, quantifiable gains in LVO/MeVO detection among VN fellows. The AR-based hologram viewing provided additional, clinically meaningful improvements particularly for territories that are harder to conceptualize using 2D viewers. The AR advantage likely reflects ‘at-a-glance’ 3D comprehension, natural spatial interaction (rotate/scale/walk-around), and reduced cognitive load compared with standard 2D scrolling. Implementing the entire Holo-PACS process, including structured education, repeated testing, and holographic visualization, resulted in significant improvement.

Given the effectiveness of NIR therapies, the necessity for stroke specialists to quickly identify LVOs/MeVOs has significantly increased [4–8]. Doing so without immediate neuroradiologist interpretation is frequent in many hyperacute stroke programs, whether patients are direct arriving LVOs (DALVO) or Brain Emergency Management Initiative (BEMI) transfers in telemedicine networks [18,19,30–32].

The American Board of Psychiatry and Neurology began VN subspecialty certification in 2005 in part to recognize additional stroke-specific educational requirements [33] There are currently only 103 accredited VN programs, resulting in a specialty shortage [34–36]. As it is critical for stroke specialists to identify LVOs, the ACGME’s patient care and procedural skills cite that fellows must demonstrate competence in neuroimaging [34]. Stroke-specific artificial intelligence (AI) tools have become commonplace to augment clinical readers’ ability to detect LVOs [37–41]. One such AI tool is now deployed throughout numerous phases of the stroke treatment and management process [31,32,42–45]. Though AI will undoubtedly augment care, it is still critical for providers to learn to identify these occlusions.

The two purposes of this Holo-PACS QI project were to assess whether targeted CTA reading education early in stroke fellowship training would result in measurable improvements in VNs’ ability to detect LVO/MeVO ‘at-a-glance’, and to determine whether the addition of the Holo-Stroke/Holo-PACS technique could be useful in overall clinical education regarding viewing vessel occlusions.

First, we noted robust improvement in assessing CTAs for LVO comparing Evaluation 1 (Group A) to Evaluation 2 (Group B). Ability to detect LVO location improved by 20% overall, which was attributable to the dedicated educational sessions. Education delivered by an experienced clinician who could detail tips and tricks to navigate real-world scenarios underscores that education, when delivered appropriately, can be immensely effective [46]. Second, we noted additional improvement in assessing for LVO when comparing Evaluation 2 (Group B) to Evaluation 3 (Group C: which used the Holo-Stroke 3D hologram technique). Ability to detect LVO location improved by 15% overall. Finally, when comparing Evaluation 1 (Group A) to Evaluation 3 (Group C: which used the Holo-Stroke 3D hologram technique) the ability to detect LVO location improved by 35% overall. The 35% overall improvement was attributed to the ‘at-a-glance’ ability to see the LVO in 3D space (resizing, rotating, or physically interacting with the realistic depiction of the 3D vessels). Providers especially noted marked improvement with vessels that are often more difficult to diagnose (e.g. PCA with 57% improvement, and ACA with 88% improvement). Utilizing the Holo-Stroke 3D hologram technique clearly resulted in significant improvement, improving to the same skill level as our stroke faculty [20].

From an educational perspective, even a single focused teaching session improved performance, underscoring the value of a structured CTA curriculum early in training. Integrating Holo-PACS into weekly case conferences was feasible and highly rated, suggesting AR can augment, not replace, standard DICOM and AI triage tools. Unlike AI software, which assists with detecting occlusions during clinical care, Holo-PACS is designed to educate the provider and improve their own ability to interpret CTA studies. Assessment of satisfaction included an overall evaluation of the Holo-PACS experience (which included both the Holo-Stroke training experience as well as the weekly incorporation of the Holo-PACS workflow in the review of clinical cases from the prior week). Our metrics were robust and consistent with our prior publications, showing a 94% overall rating and 90% overall satisfaction of Holo-PACS compared to 47% and 55% for standard DICOM technique [20]. Providers noted a high perception of immersion (96% vs. 34%), accuracy (93% vs. 55%), and enthusiasm (97% vs. 45%), with nearly all providers preferring Holo-PACS vs. DICOM technique on all metrics assessed. Whether this can eventually be used in clinical practice remains to be seen.

From an operational perspective, direct PACS integration with on-premise rendering allowed rapid, secure educational review without new PHI risks. Wider deployment will require attention to form-factor, multi-device synchronization, and remote secure access. Holo-PACS success will likely be tied to ease of use and limiting delay during routine use of this technology.

Next steps include iterating on the current design to improve the form-factor (having the visor replaced by glasses) [47]. We also plan to facilitate having groups of devices simultaneously stream the main Holo-Stroke image, with one provider manipulating the image while others can view in 3D what is being shared. We will continue to assess our fellows’ proficiency in interpreting CTAs, with the expectation that competency improves with experiences [11,48–50].

There continue to be advances in both CT and 3D image processing that can improve our ability to visualize volumes generated from standard radiographic data sets [51,52]. Future technological advancements will undoubtedly benefit holographic visualization platforms such as Holo-Stroke-CTA and Holo-PACS due to improvements in image processing, image enhancement, vessel segmentation, and image quality. We look forward to incorporating new technologies related to image processing and 3D visualization, as well as adding AI techniques.

This Holo-PACS experience has several limitations, including a limited sample size with a single fellowship class of 4 VN providers and limited DICOM case numbers. Our HOLO-PACS goal was not, in and of itself, to prove that the Holo-Stroke-CTA reading technique was better than standard DICOM technique. We more so opted to focus on the educational initiative that included training in DICOM reading as well as including the new Holo-Stroke technique of 3D hologram viewing. We note that these results are preliminary and hypothesis-generating rather than definitive evidence of educational superiority. Given the limited number of cases presented, case variability may have biased the results in any direction. Our approach to having the faculty provider who collected the cases ensure equal number of LVO and MeVOs were provided, and equally difficult cases were allocated to each group, may not have been sufficient. We plan to extend this to further groups of vascular neurology fellows over a longitudinal period of time to gain more external validity.

We note that the improvements observed in Group C cannot be attributed solely to holographic visualization since this was part of an overall training in how to improve a VN fellow’s ability to read DICOM images for vessel occlusion. Other studies have detailed more direct comparisons between LVO detection using 2D images vs. 3D Holographic imaging [20]. Those studies showed significant improvement but were a different design than this assessment of multiple elements. We are unable to determine which specific element (enhanced training in DICOM reads, access to ‘at-a-glance’ 3D hologram images, or the incorporation of hologram evaluations into weekly team meetings) made the difference. Based on our previous publications, the primary driving factor may be the greater immersive satisfaction with Holo-Stroke, though all contributors remain important [20]. Given there was also improvement noted between Evaluation 1 and 2, focused education definitely played an integral role.

In the case conferences, not all providers frequently wore the HoloLens2 HMD, because our providers were dispersed geographically to multiple locations. Sharing the 3D images via ‘screen-share’ method met the primary aim to add additional perspective not available on standard 2D DICOM technique.

Another limitation is that of longitudinal increase in knowledge that may come with becoming more familiar with CTA interpretation due to natural learning. As fellows progress, they develop increasing familiarity with CTA interpretation. Although the interval between evaluation 1 and 3 was relatively short (2–4 weeks), we cannot exclude the possibility that ongoing clinical experience in that time-period may have contributed to some of the improvement noted. As such, part of the observed improvement seen in this analysis may reflect natural education progression rather than the Holo-PACS education alone.

It is important to note that the survey/questionnaire used in this experience was an investigator-developed, non-validated satisfaction survey, purpose-built to explore user perceptions. It has been used in our prior publication assessing the satisfaction of Holo-Stroke-CTA, and has demonstrated face validity. As the survey instrument was purpose-built and not formally validated, results should not be considered to have come from a formal, psychometrically validated instrument. Based on its use in our similar prior publications and its having face validity, the tool is useful for descriptive user perceptions.

Finally, we note that in scoring Evaluations 1–3 clinical history was not provided to accompany the cases. This was done purposely to test only a single variable. It is expected that with addition of clinical history, ability to localize a vessel occlusion may increase.

The Holo-PACS QI initiative combined focused training, AR-based 3D hologram CTA review (Holo-Stroke-CTA), and direct PACS access during weekly educational case review conferences all to improve education and CTA visualization. This approach has resulted in a marked improved ability to interpret CTAs and has provided significant immersive satisfaction in image evaluation. We note that the improvements observed in Group C cannot be attributed solely to holographic visualization since this was part of an overall training in how to improve a VN fellow’s ability to read DICOM images for vessel occlusion. Other studies have detailed more direct comparisons between LVO detection using 2D images vs. 3D Holographic imaging [20]. Though significant improvement was noted, that design was different than this more global assessment of multiple elements.

Improved CTA interpretation in stroke is necessary to ensure trainees deliver quality care upon their graduation from VN fellowship programs. Holo-PACS provides a practical, focused, structured, educational framework integrating CTA instruction, 3D holographic visualization, and enterprise PACS access to augment CTA image viewing. Holo-PACS is becoming integral to our stroke education and training, and may serve as a model for vascular neurology fellowship training programs.

## Figures and Tables

**Table 1.** Holo-PACS DICOM accuracy and reliability – overall.

	VN Fellows	*p*
GROUP A: Standard DICOM vs. Gold standard [12 DICOM CTA scans]		
% Correct: Presence or absence of LVO:	68.75%	<0.001 (Group A vs. Gold Standard)
Kappa:	0.28	
% Correct: Location of LVO*:	62.50%	<0.001 (Group A vs. Gold Standard)
Kappa:	0.57	
GROUP B: Standard DICOM vs. Gold Standard [12 DICOM CTA Scans]		
% Correct: Presence or absence of LVO:	93.75%	0.241 (Group B vs. Gold Standard) 0.008 (Group A vs. Group B)
Kappa:	0.83	
% Correct: Location of LVO:	83.33%	0.001 (Group B vs. Gold Standard) 0.039 (Group A vs. Group B)
Kappa:	0.81	
GROUP C: Holo-PACS vs. Gold Standard [24 Holo-Stroke CTA Scans]		
% Correct: Presence or absence of LVO:	98.96%	1 (Group C vs. Gold Standard) 0.209 (Group B vs. Group C) <0.001 (Group A vs. Group C)
Kappa:	0.94	
% Correct: Location of LVO:	97.92%	0.477 (Group C vs. Gold Standard) 0.008 (Group B vs. Group C) <0.001 (Group A vs. Group C)
Kappa:	0.96	

Table 1 shows percent correct and reliability data. Three comparisons are shown. Top: Standard DICOM Assessment (Group A) vs. Gold Standard – Before Training. Middle: Standard DICOM Assessment (Group B) vs. Gold Standard – After Training. Bottom: Holo-PACS Assessment (Group C) vs. Gold Standard – After Holo-Stroke Training. % Correct and Kappa reliability values are shown. *p* values comparing Each Group to Gold Standard, Group A vs. B, Group B vs. C, and Group A vs. C are shown. Scores are reports for VN Fellows in aggregate only. Sample sizes (n) are shown. Percentages are reported as averages.

**Table 2.** Holo-PACS DICOM accuracy and reliability – vessel subset.

	VN Fellows	*p*
GROUP A: Standard DICOM vs. Gold standard [12 DICOM CTA scans]		
% Correct: ACA LVO	12.50%	0.002 (Group A vs. Gold Standard)
Kappa:	0	
% Correct: MCA LVO	75.00%	0.45 (Group A vs. Gold Standard)
Kappa:	0.5	
% Correct: PCA LVO	37.50%	0.031 (Group A vs. Gold Standard)
Kappa:	0.25	
% Correct: ICA LVO	75.00%	0.45 (Group A vs. Gold Standard)
Kappa:	0.67	
% Correct: Basilar LVO	100.00%	1 (Group A vs. Gold Standard)
Kappa:	1	
% Correct: Normal	75.00%	0.45 (Group A vs. Gold Standard)
Kappa:	0.5	
GROUP B: Standard DICOM vs. Gold Standard [12 DICOM CTA Scans]		
% Correct: ACA LVO	62.50%	0.2 (Group B vs. Gold Standard) 0.121 (Group A vs. Group B)
Kappa:	0.5	
% Correct: MCA LVO	100.00%	1 (Group B vs. Gold Standard) 1 (Group A vs. Group B)
Kappa:	1	
% Correct: PCA LVO	75.00%	0.45 (Group B vs. Gold Standard) 1 (Group A vs. Group B)
Kappa:	0.67	
% Correct: ICA LVO	87.50%	1 (Group B vs. Gold Standard) 0.627 (Group A vs. Group B)
Kappa:	0.83	
% Correct: Basilar LVO	87.50%	1 (Group B vs. Gold Standard) 1 (Group A vs. Group B)
Kappa:	0.75	
% Correct: Normal	87.50%	1 (Group B vs. Gold Standard) 1 (Group A vs. Group B)
Kappa:	0.75	
GROUP C: Holo-PACS vs. Gold Standard [24 Holo-Stroke CTA Scans]		
% Correct: ACA LVO	100.00%	1 (Group C vs. Gold Standard) 0.099 (Group B vs. Group C) <0.001 (Group A vs. Group C)
Kappa:	1	
% Correct: MCA LVO	93.75%	1 (Group C vs. Gold Standard) 1 (Group B vs. Group C) 1 (Group A vs. Group C)
Kappa:	0.75	
% Correct: PCA LVO	93.75%	1 (Group C vs. Gold Standard) 1 (Group B vs. Group C) 0.58 (Group A vs. Group C)
Kappa:	0.9	
% Correct: ICA LVO	100.00%	1 (Group C vs. Gold Standard) 0.627 (Group B vs. Group C) 0.037 (Group A vs. Group C)
Kappa:	1	
% Correct: Basilar LVO	100.00%	1 (Group C vs. Gold Standard) 1 (Group B vs. Group C) 1 (Group A vs. Group C)
Kappa:	1	
% Correct: Normal	100.00%	1 (Group C vs. Gold Standard) 1 (Group B vs. Group C) 0.58 (Group A vs. Group C)
Kappa:	1	

Table 2 shows percent correct and reliability data for individual vessel LVOs (MCA, ACA, PCA, ICA, Basilar) as well as normal vessels. Three comparisons are shown. Top: Standard DICOM Assessment (Group A) vs. Gold Standard – Before Training. Middle: Standard DICOM Assessment (Group B) vs. Gold Standard – After Training. Bottom: Holo-PACS Assessment (Group C) vs. Gold Standard – After Holo-Stroke Training. *p* values comparing Each Group to Gold Standard, Group A vs. B, Group B vs. C, and Group A vs. C are shown. Scores are reports for VN Fellows in aggregate only. Sample sizes (n) are shown. Percentages are reported as averages.

**Table 3.** HOLO-PACS satisfaction survey results.

	DICOM CTA review process	Holo-PACS CTA review process	*p*
Referential:			
Overall: Median [Max = 60]*	27 (IQR = 10)	58 (IQR = 4)	0.002
Percentage: (13 participants total/780 Maximum)	47.44% (370/780)	93.59% (370/780)	
Q1: Satisfaction (out of 10)*	6 (IQR = 1)	9 (IQR = 1)	0.001
Percentage: (13 participants total/130 Maximum)	55.38% (72/130)	90.00% (72/130)	
Q2: Immersion (out of 10)*	4 (IQR = 2)	10 (IQR = 1)	0.002
Percentage: (13 participants total/130 Maximum)	33.85% (72/130)	96.15% (72/130)	
Q3: Ease of Use (out of 10)*	5 (IQR = 2)	9 (IQR = 1)	0.003
Percentage: (13 participants total/130 Maximum)	53.08% (72/130)	86.92% (72/130)	
Q4: Accuracy (out of 10)*	5 (IQR = 2)	9 (IQR = 1)	0.002
Percentage: (13 participants total/130 Maximum)	54.62% (72/130)	93.08% (72/130)	
Q5: Advanced Technology (out of 10)*	4 (IQR = 2)	10 (IQR = 0)	0.002
Percentage: (13 participants total/130 Maximum)	42.31% (72/130)	98.46% (72/130)	
Q6: Interest/Enthusiasm (out of 10)*	5 (IQR = 1)	10 (IQR = 1)	0.002
Percentage: (13 participants total/130 Maximum)	45.38% (72/130)	96.92% (72/130)	
Comparative:			
Overall: Median [Max = 6]*	0 (IQR = 0)	6 (IQR = 0)	<0.001
Percentage: (13 participants total/78 Maximum)	3.58% (3/78)	96.15% (75/78)	
Q1: Which Technique is Better Overall? (out of 1)^	0 (IQR = 0)	1 (IQR = 0)	<0.001
Percentage: (13 participants total/13 Maximum)	0.00% (0/13)	100.00% (13/13)	
Q2: Which Technique is Easier? (out of 1)^	0 (IQR = 0)	1 (IQR = 0)	0.09
Percentage: (13 participants total/13 Maximum)	23.08% 3/130)	76.92% (10/13)	
Q3: Which Technique is More Effective? (out of 1)^	0 (IQR = 0)	1 (IQR = 0)	<0.001
Percentage: (13 participants total/13 Maximum)	0.00% (0/13)	100.00% (13/13)	
Q4: Which Technique Do You Prefer? (out of 1)^	0 (IQR = 0)	1 (IQR = 0)	<0.001
Percentage: (13 participants total/13 Maximum)	0.00% (0/13)	100.00% (13/13)	
Q5: Which Technique is More Technologically Advanced? (out of 1)^	0 (IQR = 0)	1 (IQR = 0)	<0.001
Percentage: (13 participants total/13 Maximum)	0.00% (0/13)	100.00% (13/13)	
Q6: Which Technique has More Future Potential? (out of 1)^	0 (IQR = 0)	1 (IQR = 0)	<0.001
Percentage: (13 participants total/13 Maximum)	0.00% (0/13)	100.00% (13/13)	

Table 3 shows provider immersion and satisfaction survey results. Both referential (6 individual questions about the Standard DICOM CTA Image Review Process, and 6 questions about the Holo-PACS CTA Image Review Process) and comparative (6 questions comparing one technique directly to the other) are shown. Overall Totals are also shown. *p* values and sample size (n) are noted. Results are shown as Median (Inter-Quartile Range) and percentages.

*: Tested using Wilcoxon Signed Rank Test.

^: Tested using Exact Binomial Test with Clopper-Pearson confidence intervals.
